# A novel in-plane technique ultrasound-guided pericardiocentesis via subcostal approach

**DOI:** 10.1186/s13089-022-00271-9

**Published:** 2022-05-21

**Authors:** Adi Osman, Azma Haryaty Ahmad, Nurul Shaliza Shamsudin, Muhammad Faiz Baherin, Chan Pei Fong

**Affiliations:** 1 Consultant Emergency Physician & ED Critical Care, Resuscitation & Emergency Critical Care Unit (RECCU), Trauma & Emergency Department, Hospital Raja Permaisuri Bainun, Ipoh, Perak Malaysia; 2 Emergency Physician, Trauma & Emergency Department, Hospital Raja Permaisuri Bainun, Ipoh, Perak Malaysia

**Keywords:** Pericardiocentesis, Ultrasound-guided, Subcostal Approach, Needle Visualization

## Abstract

**Background:**

Cardiac tamponade occurs when fluid or blood, fills the pericardial space, and causes hemodynamic compromise due to compression of the heart. It is a potentially life-threatening condition, that requires rapid recognition and immediate treatment. Formerly, blind or surgical techniques were used, and it is associated with complications. Medical technology development has enabled us to perform the procedure safely, with the assistance of ultrasound devices. This article will highlight the novel use of an in-plane subcostal technique, as a safe option for pericardiocentesis in cardiac tamponade.

**Case presentation:**

A 50-year-old man presented to the emergency department (ED) with shortness of breath and shock. He was intubated for respiratory distress. His bedside echocardiography showed cardiac tamponade. Ultrasound-guided pericardiocentesis was carried out using an in-plane technique, at the subcostal region, with a high-frequency linear ultrasound transducer. This particular method provided full visualization of needle trajectory throughout the procedure. It was successfully completed with no complications and patient’s hemodynamic status improved post-procedure. He was successfully discharged on day 13.

**Conclusions:**

The in-plane subcostal pericardiocentesis is a safe, and simple approach that can be performed in the ED for patients with cardiac tamponade. We recommend this new in-plane method, with high-frequency linear transducer at the subcostal area as an alternative when cardiac window for other approaches cannot be visualized.

**Supplementary Information:**

The online version contains supplementary material available at 10.1186/s13089-022-00271-9.

## Background

Cardiac tamponade is a life-threatening clinical condition caused by rapid accumulation of pericardial fluid, resulting in impaired ventricular filling, decreased cardiac output, and hemodynamic instability [1]. Prompt recognition and urgent intervention to treat cardiac tamponade is life-saving.

Ultrasound-guided pericardiocentesis is currently considered the gold-standard for pericardial fluid aspiration. This technique was first introduced in 1979, and had become the preferred technique for cardiac tamponade management [2]. This technique had been proven to be effective, and had lower risks of complications compared to blind or surgical techniques [2, 3].

Since its first introduction, the procedure of ultrasound-guided pericardiocentesis, had been refined into better techniques with different approaches [4]. In the early days, echocardiography was performed with a low-frequency transducer to diagnose pericardial effusion and locate the best site for puncture [5]. The older practice of using echocardiography assistance, did not provide continuous ultrasound visualization of needle trajectory, and had a complication rate of about 5% [10]. The newer approach, is a true echocardiography-guided procedure that uses a high-frequency transducer to track the needle in real time, allowing the clinician to avoid injury to the surrounding structures [5, 7].

This is a case report of our experience with a novel subcostal in-plane ultrasound-guided pericardiocentesis, using linear transducer for a patient with cardiac tamponade at the emergency department (ED).

## Case presentation

A 50-year-old man with underlying hypertension, presented to the ED with shortness of breath for 1 week. He was conscious on arrival, but restless, and sitting in a tripod position. His vital signs revealed a blood pressure of 85/45 mmHg, heart rate of 120 beats per minute, respiratory rate of 28 breath per minute, temperature of 37 C, and oxygen saturation (SpO_2_) of 82% with oxygen 12 l/min. Physical examination revealed he had labored breathing with usage of respiratory accessory muscles, and he was diaphoretic. His jugular venous pressure was not raised and his cardiorespiratory system did not reveal any significant findings. He was intubated and mechanically ventilated. His electrocardiogram showed sinus tachycardia, and his chest radiograph revealed cardiomegaly with right pleural effusion. A bedside point-of-care echocardiography showed hyperdynamic left ventricle, with massive pericardial effusion. Right ventricular collapse was also noticed during diastole and collectively, this was indicative of a cardiac tamponade (Additional file 1: Video S1).

**Fig. 1:**
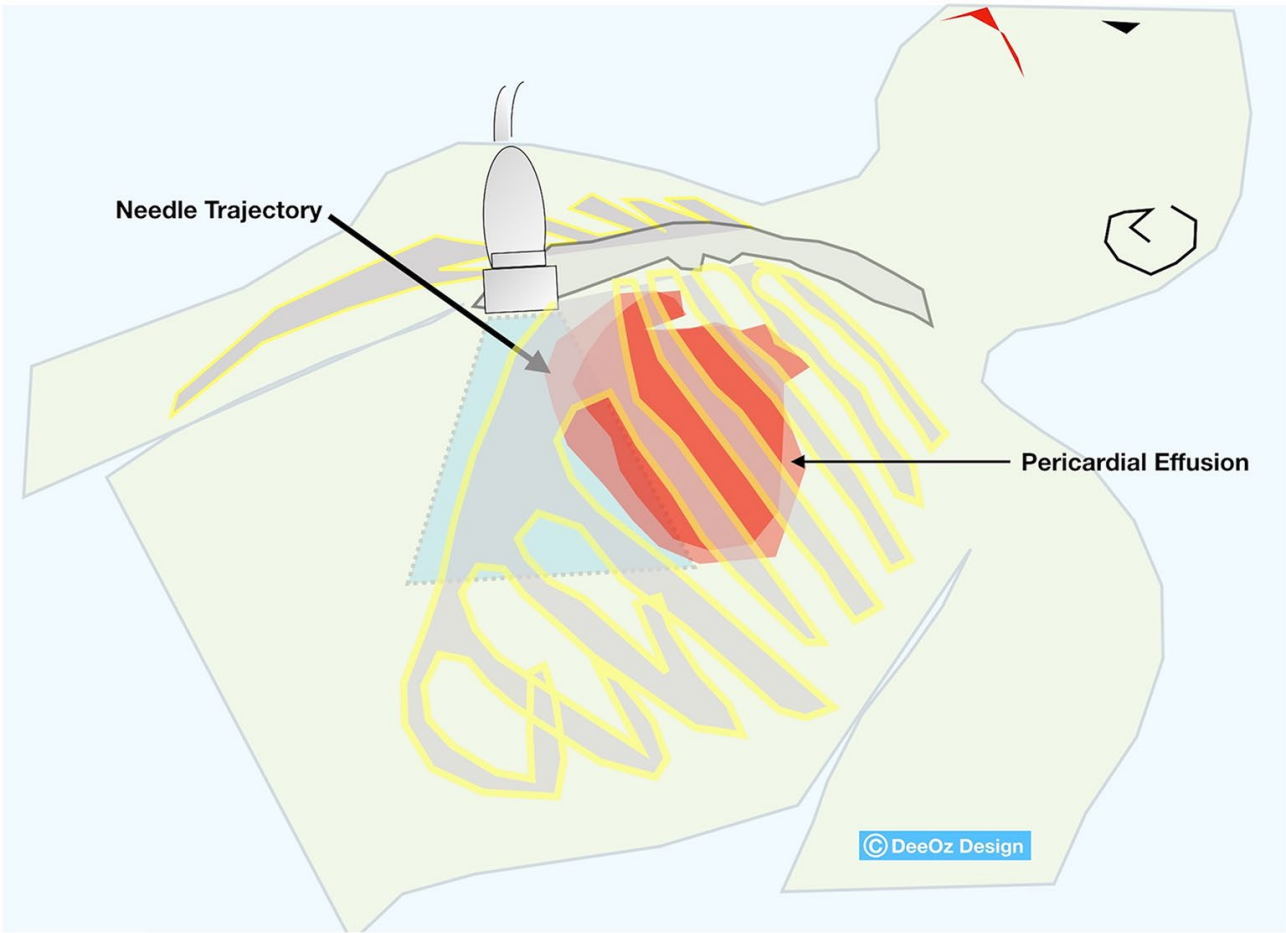
High-frequency linear ultrasound transducer was placed horizontally at subcostal area with the marker pointing caudally

**Fig. 2:**
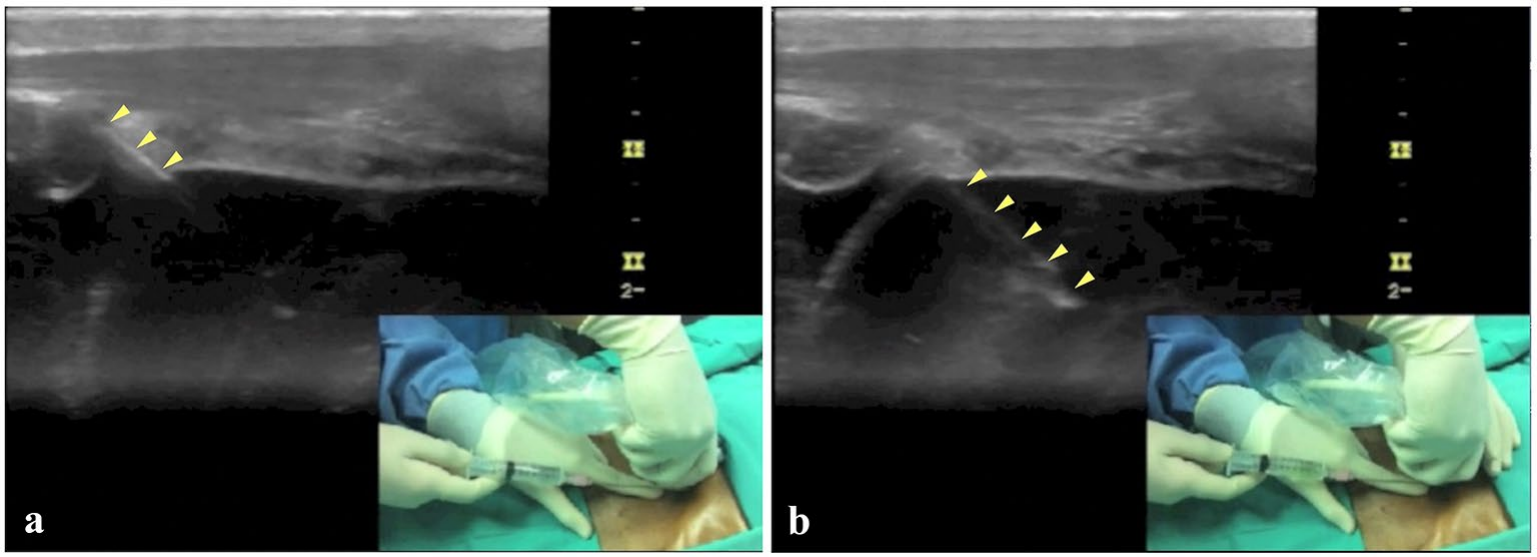
**a** Needle tip was visualized piercing the pericardial sac, **b** using in-plane technique, the needle tip was fully visualized as it was advanced into the pericardial space. 200 ml of hemoserous fluid was aspirated and a pigtail catheter was left in-situ for continuous drainage

Pericardiocentesis was done by the emergency physician in-charge under ultrasound guidance using subcostal approach. A high-frequency linear ultrasound transducer was placed horizontally at subcostal area with the marker pointing caudally. Using in-plane technique, the needle tip was fully visualized as it was advanced into the pericardial space. 200 ml of hemoserous fluid was aspirated and a pigtail catheter was left in-situ for continuous drainage (Figs. 1, 2 and Additional file 2: Video S2). Post procedure, his blood pressure improved to 130/90 mmHg, he became less tachypneic and his heart rate improved to 100 beats per minute.

The pericardial fluid cytology revealed malignant epithelial cells exhibiting large pleomorphic nuclei. A computed tomography of his whole body showed multiple metastatic lesions of the lungs, liver, right adrenal and pelvic region with unknown source of malignancy. He was ventilated in intensive care unit for 13 days, and was referred to palliative care team upon discharged.

## Discussion

This case illustrated a successful pericardiocentesis procedure using subcostal approach, with real-time ultrasound guidance. The contemporary use of ultrasound has allowed pericardiocentesis to be performed at any position surrounding the pericardium [4, 5]. In this case, the subcostal site was chosen, because this was where the image was clearest, and the pericardial collection was largest.

Before the era of ultrasound, subxiphoid or subcostal approach, was the most widely accepted method, due to its high success rate to locate anatomical landmark at Larrey’s triangle [8, 9]. After the introduction of ultrasound, the practice had changed tremendously, and anatomical location for pericardiocentesis varies. Para-apical had been found to be the most common site (63%), followed by subcostal (15%) and parasternal (14%) [6]. The para-apical approach was preferred, because it was usually, where the pericardial space was closest to the probe, and the fluid accumulation was maximal [5, 10].

Osman et al. demonstrated that the left parasternal with medial to lateral approach could provide an excellent visualization of needle trajectory [11]. Under ultrasound guidance, the left parasternal approach avoids injury to the surrounding structures, making the procedure practically free from any complications.

When choosing the site of the emergency pericardiocentesis, the ideal approach for using point-of-care ultrasound guidance should take into consideration the distance of effusion to the probe, image quality, and predicted complications. Stolz et al. predicted that the subcostal approach had the highest complication rate compare to other methods [12]. This was because of its long distance from the skin to the pericardial space, which increased the risk of injury to the liver, blood vessels and bowels. However, subcostal approach might be the preferred option in situations, such as cardiopulmonary resuscitation, or poor view for other approaches due to hyperinflated lungs.

When pericardiocentesis was performed blindly using the subcostal approach, it had a complication rate of 5–20% [6, 8]. In the year 2000, Vayre et al. reported 109 cases of ultrasound-guided subcostal pericardiocentesis with contrast study [13]. However, a 10% rate of right ventricular puncture was still observed. The author concluded that although pericardial contrast injection could help to localize the needle tip, it did not prevent traumatic punctures. This was probably because the procedure was done using a low-frequency phased array transducer, and it was not under true real-time ultrasound guidance [14]. 

Recently, Law et al. demonstrated that the subcostal approach could still be a safe procedure. He confirmed this using long axis in-plane technique at the subcostal area for pericardiocentesis. The procedure was carried out on 14 post-operative pediatric patients and no complications were observed [15]. In adult patients, the increase of depth of surrounding tissues and structures may affect angulation of the needle and it will be more challenging. In this case, we demonstrated that the in-plane subcostal approach using high-frequency linear probe was feasible in an adult patient.

## Conclusions

The in-plane subcostal pericardiocentesis is a safe and simple approach that can be performed in ED for patients with cardiac tamponade. We recommend this new technique as an alternative when cardiac window for other approaches cannot are not possible.

## Supplementary Information

**Additional file 1. **Bedside point of care echocardiography showed hyperdynamic left ventricle with massive pericardial effusion. Right ventricular collapse was also noticed during diastole and collectively, this was suggestive of cardiac tamponade.

**Additional file 2. **A high frequency linear ultrasound transducer was placed horizontally at subcostal area with the marker pointing caudally. Using in-plane technique, the needle tip was fully visualized as it was advanced into pericardial space. 200 ml of hemoserous fluid was aspirated and a pigtail catheter was left in-situ for continuous drainage.

## Data Availability

The material are available from the corresponding author on reasonable request.

## Abbreviations

ED: Emergency Department
SpO2: Oxygen saturation
